# Analysis of *Drosophila p8* and *p52* mutants reveals distinct roles for the maintenance of TFIIH stability and male germ cell differentiation

**DOI:** 10.1098/rsob.160222

**Published:** 2016-10-19

**Authors:** Grisel Cruz-Becerra, Mandy Juárez, Viviana Valadez-Graham, Mario Zurita

**Affiliations:** Departamento de Genética del Desarrollo, Instituto de Biotecnología, Universidad Nacional Autónoma de México, Av Universidad 2001, Cuernavaca Morelos 62250, Mexico

**Keywords:** TFIIH, transcription, cell differentiation, spermatogenesis, meiotic arrest, *Drosophila*

## Abstract

Eukaryotic gene expression is activated by factors that interact within complex machinery to initiate transcription. An important component of this machinery is the DNA repair/transcription factor TFIIH. Mutations in TFIIH result in three human syndromes: xeroderma pigmentosum, Cockayne syndrome and trichothiodystrophy. Transcription and DNA repair defects have been linked to some clinical features of these syndromes. However, how mutations in TFIIH affect specific developmental programmes, allowing organisms to develop with particular phenotypes, is not well understood. Here, we show that mutations in the p52 and p8 subunits of TFIIH have a moderate effect on the gene expression programme in the *Drosophila* testis, causing germ cell differentiation arrest in meiosis, but no Polycomb enrichment at the promoter of the affected differentiation genes, supporting recent data that disagree with the current Polycomb-mediated repression model for regulating gene expression in the testis. Moreover, we found that TFIIH stability is not compromised in p8 subunit-depleted testes that show transcriptional defects, highlighting the role of p8 in transcription. Therefore, this study reveals how defects in TFIIH affect a specific cell differentiation programme and contributes to understanding the specific syndrome manifestations in TFIIH-afflicted patients.

## 1. Introduction

Proliferation and cell differentiation are linked to cell cycle modulation, global gene expression and genome maintenance. Several factors mediate the crosstalk among these mechanisms. One of these is the DNA repair and basal transcription factor TFIIH, which participates in transcription with RNA polymerases I and II (RNAPI and RNAPII), nucleotide excision repair (NER) and cell cycle regulation in metazoans [1,2]. TFIIH is composed of a core and cyclin-dependent activating kinase (CAK) subcomplexes. The core includes XPB and XPD as well as the p62, p52, p44, p34 and p8 subunits. Cdk7, cyclin H and MAT1 constitute the CAK subcomplex [3]. Several enzymatic activities have been identified in components of the TFIIH complex. Recently, a DNA translocase activity was attributed to XPB [4]. In addition, XPB and XPD are DNA helicases/ATPases essential for transcription and DNA repair, while Cdk7 is one of the major kinases involved in transcription activation and cell cycle regulation [5,6]. On the other hand, most TFIIH subunits, including p52, p8, p34, Cyc H and Mat1, have been described as key modulators of these enzymatic activities [7–9]. Additionally, a main role in maintaining steady-state TFIIH levels has been attributed to the p8 subunit [10].

Mutations in TFIIH are associated with complex human diseases, including cancer. Mutations in the XPB and XPD subunits may cause xeroderma pigmentosum (XP), Cockayne syndrome (CS) and trichothiodystrophy (TTD) [11]. In the case of p8, the different mutations described in humans have been related only to TTD-A, which is caused by reduced TFIIH levels in p8 mutant cells [10]. Cutaneous photosensitivity is a shared feature among these syndromes, but other manifestations are syndrome-specific. For example, XP patients may develop skin cancer. TTD is characterized by mental and physical retardation, sterility, ichthyosis and brittle hair. By contrast, CS is characterized by premature ageing, growth failure and progressive neurological dysfunction [12]. As expected, some clinical symptoms in XP, CS and TTD patients have been strongly linked to DNA repair defects as well as transcriptional deficiencies [13]. In this regard, it is intriguing how individuals affected by a general transcription factor that also has a role in cell cycle control and NER are able to complete development showing very specific phenotypes. This may be explained by considering that the mutations observed in human patients are not null, but rather partially affect TFIIH functions [13,14]. Additionally, specific cell types or specific gene expression programmes could be more susceptible to these defects. For example, male sterility is a common feature among several TFIIH mutants in *Drosophila* [15–17] and some TFIIH-afflicted patients [2]. Therefore, the study of how TFIIH mutations affect global transcription in different cell types and how this may affect proliferation or differentiation programmes during the development of model organisms will contribute to an understanding of the basis of the clinical features demonstrated by TFIIH-afflicted patients.

*Drosophila* spermatogenesis consists of a multi-step differentiation programme that involves easily observable cellular morphology changes and a well-defined gene expression programme that allows stem cells to become highly specialized sperm cells in the testis. Germ cell differentiation requires the transcriptional activation of approximately 2000 genes in the *Drosophila* testis [18]. The model proposed to explain how germ cell differentiation is achieved involves the participation of testis-specific TBP-associated factors (tTAFs) and some components of the testis-specific meiotic arrest complex (tMAC), which are encoded by some meiotic arrest genes [19] that positively regulate the expression of their targets by interacting with the mediator complex and by sequestering Polycomb (Pc) in a particular compartment of the nucleolus to counteract the repression of differentiation genes in the primary spermatocyte stage during differentiation [19–21]. Here, we report that mutations in the core subunits of the general transcription factor TFIIH generate a meiotic arrest phenotype similar to that observed in testis-specific TAF mutants. A TFIIH mutation delocalizes Pc from the nucleolus in primary spermatocytes. However, Pc binding is not enhanced at the promoter of the repressed differentiation genes in the TFIIH mutant testes, which supports recent genome-wide data that challenge the participation of Pc in the repression of tTAFs targets [22]. Interestingly, our data show that mutations in the p8 and p52 subunits of TFIIH do not affect the transcription of most genes in the *Drosophila* testis. Instead, genes required for terminal differentiation, but not their testis-specific transcriptional regulators, are downregulated, suggesting a gene-specific requirement for TFIIH in transcription during this cell differentiation programme. Furthermore, contrary to the effects of the mutations in p8 observed in cells from TTD-A patients [10], the analysis of p8-depleted testes, which showed transcriptional defects, revealed that the stability of other TFIIH subunits is not compromised, highlighting a role for p8 in transcription beyond its role in TFIIH stability maintenance.

## 2. Material and methods

### 2.1. Fly stocks

*Ore R* was used as wild-type strain (*p8^+^*/*p8^+^* and *p52^+^/p52^+^*), except when indicated. The *Pc-GFP* transgenic line (BL9593) was obtained from the Bloomington Stock Center. The *p52^EP3605^*, *p52^mrn^5^^*, *p52^mrn^3^^* and *p8^−^* alleles were previously described [15,16].

### 2.2. Phase-contrast and confocal microscopy

Testes from 0 to 1 day post eclosion were dissected in testis buffer [23] and examined by phase-contrast and confocal microscopy. Visualization of fluorescently tagged proteins was performed using the Zeiss LSM 510 META confocal system coupled to an Axiovert 200 inverted microscope.

### 2.3. Transgenic constructs

DNA recombinant constructs of p8-ECFP, XPB-EGFP and EYFP-p52 were generated by tagging the full-length open reading of p8, p52 and XPB, in frame with the DNA sequence of the Enhanced-Cyan, Enhanced-Green or the Enhanced-Yellow Fluorescent Proteins. These constructs were cloned into the pCaSper-Hsp83 vector.

### 2.4. Rescue experiments

Rescue of the semi-lethality phenotype of homozygous *p8* mutant flies was performed by crossing heterozygotes (*p8^+^/p8^−^*) mutants with pCaSper-Hsp83 transgenic lines expressing the p8-ECFP recombinant protein. The F_1_ progeny was intercrossed to generate homozygous *p8^−^/p8^−^* flies containing one or two copies of the p8-ECFP transgene. Similar crosses were performed to rescue the semi-lethality phenotype of the heteroallelic combination of *p52* mutants (*p52^EP3605^*/*p52^mrn^5^^*) with transgenic lines expressing EYFP-p52.

### 2.5. Western blotting

Testes were dissected in ice-cooled PBS with protease inhibitors (complete, Roche). Total protein extracts were analysed by immunoblotting using standard procedures. Primary antibodies used were: 8WG16 (1 : 1500; Covance), H14 (1 : 1500; Covance), XPD (1 : 1500; our own preparation), XPB (1 : 2000; our own preparation), p52 (1 : 1500 [16]), p8 (1 : 1000 [16]), Cdk7 (1 : 1000, Santa Cruz Biotechnology), TBP (1 : 500; Santa Cruz Biotechnology), E7 (1 : 2000; DSHB), A12 (1 : 1500; DSHB), F2F4 (1500; DSHB), JLA20 (1 : 3000; DSHB), GFP (1 : 2500; GenScript); p18 (1 : 1000; [16]) HRP-coupled secondary antibodies (1 : 3500; Invitrogen) were used for chemioluminiscence detection with Thermo Scientific Pierce ECL. Densitometric analyses in western blots were performed using ImageJ software. Protein levels among the different genotypes were normalized to TBP and quantified with respect to the correspondent wild-type (*p8^+^*/*p8^+^* and *p52^+^/p52^+^*genotypes) protein amount set to 1.

### 2.6. qPCR expression analysis

Total RNA from testes was extracted with Trizol (Invitrogen). Equal quantity of RNA from each genotype was used to synthetize cDNA with MVL-V reverse transcriptase (Invitrogen). qPCR analyses were performed with LightCycler Fast Start DNA Master^PLUS^ SYBR Green I and the LightCycler 1.5 Instrument (Roche). The relative expression level of each analysed gene was calculated by 2^−ddCt^, where ddCt = (Ct target gene-Ct control gene) using *CycA* as internal control. Primer sequences (5′-3′) for *Mst87F*: forward, aacttttacgaattaatcatgtgctg; reverse, cagggtccacatcctcctc. *dj*: forward, aactgaaaaagaaatgcaaggaa; reverse, tttgcaagggtctttcttcg. *fzo*: forward, caatgtctctccatacccctaca; reverse, agttgccaatcgcaagagtt. *twe*: forward, aagaccaagtcctggcaatg; reverse, cagtcgtgaacgtgatttcc. *CycA*: forward, gctggaggagatcacgactt; reverse, ccatcatagccaccttcttgt.

### 2.7. Chromatin immunoprecipitation-qPCR

Chromatin immunoprecipitation from testes was performed as reported previously [20] with small modifications. Briefly, 100 pairs of testes were used per assay. Cross-linking was performed with 1% formaldehyde in PBS for 15 min at 37°C, followed by washing with PBS. Testes were disrupted in 130 µl of SDS-lysis buffer (1% SDS, 50 mM Tris-HCl, pH 8.0, 10 mM EDTA) and sonicated in thin wall 0.6 ml tubes (Axygen PCR-05-C) for 6 cycles (1 cycle is: 30 s ON/60 s OFF) at high setting using a Diagenode Bioruptor. Chromatin was diluted 1 : 10 with ChIP dilution buffer (0.01% SDS, 1.1% Triton X-100, 1.2 mM EDTA, 16.7 mM Tris-HCl, pH 8.0, 167 mM NaCl) and pre-cleared with rabbit IgG coupled to Dynabeads Protein G (Life Technologies). After pre-clearing, 10% of the lysate was reserved as input. The lysate was incubated with 7.5 µg of the antibody (anti-Pc or anti-XPB; Santa Cruz Biotechnology) or irrelevant rabbit IgG (Invitrogen) overnight at 4°C. The antibody-chromatin complexes were pulled-down with 50 µl of Dynabeads Protein G. Beads were washed once with low-salt wash buffer (0.1% SDS, 1% Triton X-100, 2 mM EDTA, 20 mM Tris-HCl, pH 8.0 and 150 mM NaCl), once with high-salt wash buffer (0.1% SDS, 1% Triton X-100, 2 mM EDTA, 20 mM Tris-HCl, pH 8.0 and 500 mM NaCl), once with LiCl wash buffer (0.25 M LiCl, 1% NP40, 1% sodium deoxycholate, 1 mM EDTA, and 10 mM Tris-HCl, pH 8.0) and twice with TE (10 mM Tris-HCl, pH 8.0 and 1 mM EDTA). Immunoprecipitated chromatin was eluted with elution buffer (1% SDS, 0.1 M NaHCO_3_ in 1xTE). Reverse cross-linking was performed at 65°C, followed by RNA and protein digestion. The DNA was recovered by phenol/chloroform extraction and ethanol/glycogen precipitation. Immunoprecipitated DNA was analysed by qPCR, using the primers reported previously [21,22]. The fold enrichment relative to mock was calculated by *E*^(−ddCt)^, where *E* represents the efficiency of each gene primers, and ddCt = [(Ct sample − Ct input) − (Ct mock − Ct input)] [24,25].

### 2.8. RNA-seq and bioinformatics analysis

Total RNA from *Ore R*, *p8^−^/p8^−^* and *p52^EP3605^/p52^mrn^5^^ *testes were prepared with trizol. The Beijing Genomic Institute (BGI) performed the RNA-seq and part of the data analysis. In summary, libraries for RNA sequencing were prepared with poly-A-selected mRNA using the Illumina TruSeq RNA library construction kit v2. Libraries were purified using the Agencourt AMPure XP (Beckman Coulter) and run as 50 bp single-end lanes on an Illumina HiSeq 2000 instrument. To test the gene expression enrichment, BGI examined the reads per kilobase transcriptome per million mapped reads (RPKM) [26] values in the different samples. Correction for false positive and false negative errors was performed by using the FDR method [27].

### 2.9. Transcriptome comparison between TFIIH mutants and other meiotic arrest mutants

The significant log_2_-fold change values for the DEGs in the transcriptomes of *sa, aly, Med22* and *Ubi-p63E* were obtained from the data in the GEO database with the GEO accession numbers previously reported [19,28,29]. In the case of the *bam* transcriptome, the log_2_-fold change values were calculated from the reported RPKM data for each analysed gene [30].

## 3. Results

### 3.1. TFIIH core subunits are required for meiotic divisions during spermatogenesis

Similar to humans, mutations in TFIIH subunits in the fly cause complex and pleiotropic phenotypes [31]. We have previously reported a *p8* gene null allele (*p8^−^*), which is semilethal, though the males are sterile [16]. Semi-lethality and sterile organisms were also observed from heteroallelic combinations between a P-element insertion allele (*p52^EP3605^*) and point mutation alleles (*p52^mrn^3^^* and *p52^mrn^5^^*) in the p52 subunit of TFIIH [15]. To better understand the function of TFIIH during cell differentiation, we investigated the cellular and molecular processes affected during spermatogenesis in the *p8* and *p52* mutant testes.

In wild-type organisms, spermatogenesis is initiated at the apical tip of the testis with the asymmetrical division of a stem cell to produce a daughter stem cell and a spermatogonial precursor cell, which after four rounds of mitotic divisions undergoes premeiotic S phase and becomes a 16-primary spermatocyte cyst. At the primary spermatocyte stage, the transcriptional programme required for the meiotic divisions and terminal differentiation is activated, allowing postmeiotic spermatid differentiation [18].

**Figure 1.**
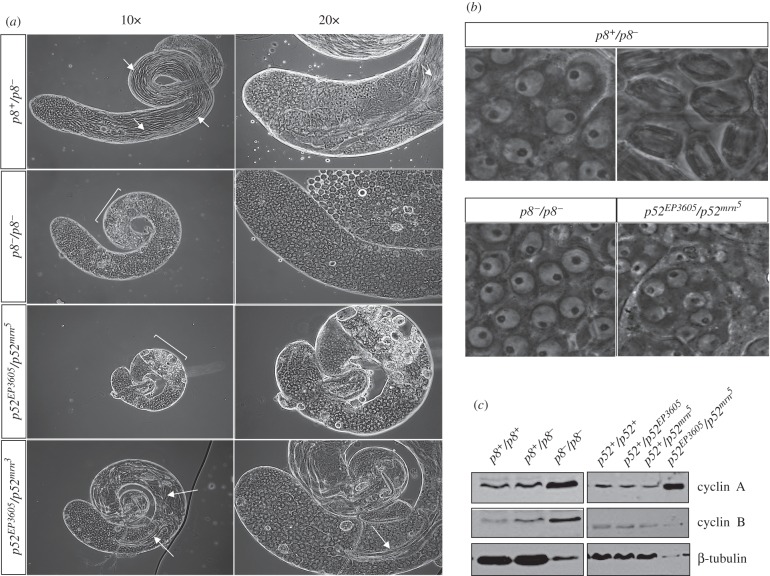
Meiotic arrest phenotype in *p8* and *p52* mutant testes. (*a*) Phase contrast of squash testes from heterozygous *p8* mutant (*p8^+^/p8^−^*), homozygous *p8* mutant (*p8^−^/p8^−^*) and heteroallelic *p52* mutants (*p52^EP3605^/p52^mrn^5^^* and *p52^EP3605^/p52^mrn^3^^*). Note that all the TFIIH mutant testes are smaller than control (*p8^+^/p8^−^*): *p8^−^/p8^−^*, *p52^EP3605^/p52^mrn^5^^*and *p52^EP3605^/p52^mrn^3^^* testes represent about 0.67×, 0.3× and 0.8× the wild-type size, respectively. The *p8^+^/p8^−^* testes show all the stages of spermatogenesis and flagella bundles (small arrows) are easily observed. By contrast, *p8^−^/p8^−^* and *p52^EP3605^/p52^mrn^5^^* testes are filled with primary spermatocytes and some degenerating cells (square bracket). Similarly, the *p52^EP3605^/p52^mrn^3^^* testes are mostly enriched with primary spermatocytes; however, some bundles of flagella (big arrows) are also observed. (*b*) Primary spermatocytes at metaphase of meiosis I are observed in control (*p8^+^/p8^−^*, top right panel), but not in *p8^−^/p8^−^* or *p52^EP3605^/p52^mrn^5^^* testes. (*c*) Western blots for the indicated proteins in *p8^−^/p8^−^*, *p52^EP3605^/p52^mrn^5^^* and control (*p8^+^/p8^+^*, *p8^+^/p8^−^*, *p52^+^/p52^+^*, *p52^+^/p52^mrn^5^^* and *p52^+^*/*p52^EP3605^*) testes. The same number of testes was loaded for each genotype.

To gain insights into the *p8^−^/p8^−^*, *p52^EP3605^/p52^mrn^3^^* and *p52^EP3605^/p52^mrn^5^^* male sterility phenotypes, we analysed their testes by phase-contrast microscopy. We noticed that TFIIH mutant testes were smaller than wild-type (figure 1*a*). Furthermore, squash preparations from live mutant testes showed an arrest of germ cell differentiation at the primary spermatocyte stage, while groups of degenerating cells were observed at the base of the testes (figure 1*a*). Unlike wild-type primary spermatocytes, which showed nucleolus break down and metaphasic spindle assembly at meiosis I, the TFIIH mutant primary spermatocytes failed to enter meiosis, arresting at the G2/M transition (figure 1*b*). Although these phenotypes were similar between the mutant testes from the two different TFIIH subunits, we observed an effect on the severity of the phenotype that depended on the allele combination in the *p52* mutant organisms. For example, despite primary spermatocyte enrichment, some flagella bundles were observed in the *p52^EP3605^/p52^mrn^3^^* testes (figure 1*a*), indicating that spermatogenesis proceeded aberrantly in some cells even though it was not successfully completed. Moreover, the *p52^EP3605^/p52^mrn^3^^* mutant shows the less dramatic effect on testis size (i.e. 0.2× smaller than wild-type). On the other hand, the strongest phenotype was observed in the *p52^EP3605^/p52^mrn^5^^* males, which showed the smallest testis size (representing only about one-third of the wild-type size) and were filled with early primary spermatocytes (figure 1*a*). In agreement with the accumulation of premeiotic primary spermatocytes, the protein levels of some cell cycle regulators, such as cyclin A (Cyc A) and cyclin B (Cyc B), were highly increased in the *p8^−^/p8^−^* testes, while only Cyc A was increased in the *p52^EP3605^/p52^mrn^5^^* testes (figure 1*c*), suggesting that primary spermatocytes arrested at different stages during meiosis in *p8^−^/p8^−^* and *p52^EP3605^/p52^mrn^5^^* testes. Altogether these data indicate that the absence or reduction of some TFIIH proteins resulted in a meiotic arrest phenotype that impairs primary spermatocyte differentiation. Intriguingly, these phenotypes were very similar to those observed in mutant testes from the meiotic arrest genes, which only produce primary spermatocytes that fail to continue meiosis or spermatid differentiation. Most meiotic arrest genes encode testis-specific transcription factors, including tTAFs, and some components of the tMAC, which are required for the activation of several genes during differentiation [19].

### 3.2. The core subunits of TFIIH are located in the nucleus and the nucleolus periphery of primary spermatocytes

The localization pattern of several meiotic arrest gene products during spermatogenesis has been shown to be relevant to their function. For example, tTAFs are poorly detected in euchromatin but are enriched in the nucleolus of primary spermatocytes, where they have been proposed to sequester Pc to counteract Pc-mediated repression of terminal differentiation genes [20]. By contrast, *tMAC* gene products are mainly located in the bivalent chromosomes of primary spermatocytes, suggesting a major role in transcriptional activation [32,33].

**Figure 2.**
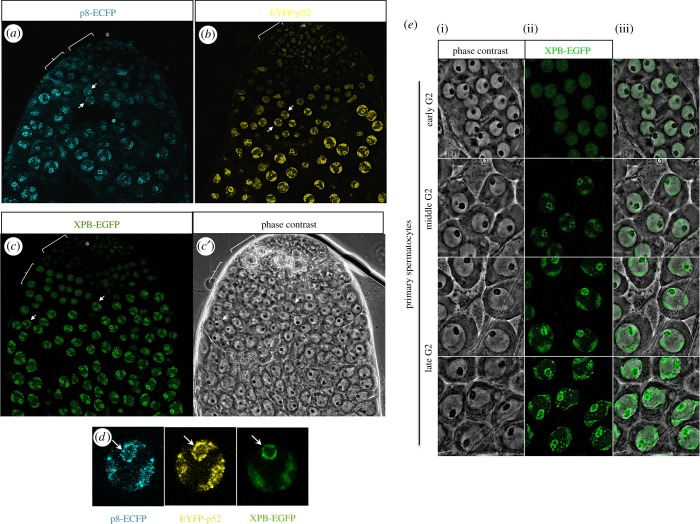
p8, p52 and XPB are enriched in primary spermatocytes. Confocal microscopy images from (*a*) p8-ECFP, (*b*) EYFP-p52 and (*c*) XPB-EGFP in the apical tip of testes. Low levels of these proteins are detected in very early germ cells and gradual enrichment is observed at the primary spermatocyte stage. (*c*′) Phase-contrast image from the XPB-EGFP testis. Asterisk: somatic cells and stem cells niche; square bracket: spermatogonium cells zone; curly brackets: very early spermatocytes zone; arrows: early spermatocytes. (*d*) p8-ECFP, EYFP-p52 and XPB-EGFP are localized at the periphery of the nucleolus (arrows) and the autosomal bivalents in mature primary spermatocytes. Images from p8-ECFP and EYFP-p52 correspond to the same primary spermatocyte obtained from testes expressing both proteins. (*e*) Dynamic of XPB-EGFP in primary spermatocytes. At the earliest stage of primary spermatocytes, XPB-EGFP is homogeneously distributed through the nucleus. As spermatocytes advance in G2, the XPB-EGFP distribution switches to particular enriched regions. In middle G2 spermatocytes, XPB-EGFP shows a foci pattern at the nucleolar periphery and it is enriched in the somatic bivalents. In mature spermatocytes, the localization of XPB-EGFP at the nucleolus periphery become ring-shaped, the enrichment at the bivalents is enhanced and foci enrichment in the nucleoplasm is observed. Panel (i) corresponds to phase-contrast images, (ii) corresponds to XPB-EGFP signal and (iii) shows the merge between them.

Because we observed that TFIIH mutant testes showed a meiotic arrest phenotype, we investigated the distribution of TFIIH during spermatogenesis. We generated transgenic flies expressing p8-ECFP or EYFP-p52. Importantly, these recombinant proteins partially rescued the *p8* and *p52* semilethal mutants, respectively (see the electronic supplementary material, tables S1 and S2). Using confocal microscopy on unfixed squashed testes from these transgenic flies, we observed restricted localization of these TFIIH core subunits to the early stages of spermatogenesis (figure 2*a,b*). Accordingly, an identical pattern for the XPB subunit was observed in the testes of transgenic flies expressing XPB-EGFP (figure 2*c*). There was also a weak signal and a homogeneous distribution in the nucleus of spermatogonial cells, but a more dynamic pattern during the spermatocyte stage (figure 2*a–c*,*e*). TFIIH fluorescently tagged proteins were enriched at bivalent chromosomes and showed a ring-shaped foci pattern at the nucleolus periphery in early spermatocytes (figure 2*d*,*e*). Later, an additional foci pattern was also observed throughout the nucleoplasm of mature spermatocytes (figure 2*e*). Thus, the localization pattern of the TFIIH subunits correlated with the stages of active transcription required to conduce germ cell differentiation in the testis.

### 3.3. The reduction of p52 levels, but not the ablation of p8, affects the stability of other TFIIH components in the *Drosophila* testis

**Figure 3.**
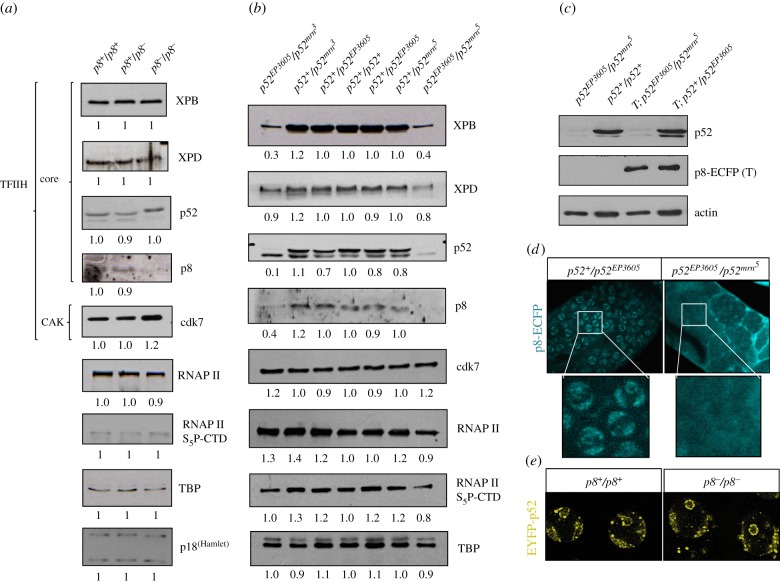
Effect on the stability of TFIIH subunits in *p8* and *p52* mutant testes. (*a*) Western blots from wild-type (*p8^+^/p8^+^*), heterozygous (*p8^+^/p8^−^*) and homozygous (*p8^−^/p8^−^*) *p8* mutant testes. (*b*) Western blots from wild-type (*p52^+^/p52^+^*)*,* heterozygous (*p52^+^/p52^EP3605^*, *p52^+^/p52^mrn^3^^* and *p52^+^/p52^mrn^5^^*) and heteroallelic combinations (*p52^EP3605^/p52^mrn^3^^* and *p52^EP3605^/p52^mrn^5^^*) of *p52* mutant testes. Note that the heteroallelic *p52* mutants show affected levels of several components of TFIIH. Densitometric analyses were performed using TBP as a loading control; the relative quantification is indicated under each blot. (*c*) Western blots in heterozygous (*p52^+^/p52^EP3605^*) and heteroallelic (*p52^EP3605^/p52^mrn^5^^*) *p52* mutant testes expressing the *p8-ECFP* transgene show no difference in p8-ECFP protein levels. The *p8-ECFP* transgene is indicated as a *T* in the genotypes. (*d*,*e*) Localization of EYFP-p52 and p8-ECFP recombinant proteins in *p8* and *p52* mutant primary spermatocytes, respectively.

The strength of the defect severity showed by the *p52^EP3605^/p52^mrn^5^^* mutant in spermatogenesis suggests that mutations in *p52* have a more deleterious effect on TFIIH functions than the absence of *p8* in the fly testes. Interestingly, the p8 mutant alleles reported in TTD-A afflicted-humans are linked to a reduction in the basal levels of TFIIH [10]. However, recent reports revealed that none of the p8 mutations found in humans are null [14,34]. To determine whether the spermatogenesis defects observed in the *p8* and *p52* mutants are related to globally reduced levels of TFIIH components, we analysed the effect of the absence of p8 or the mutation of p52 on the stability of other TFIIH subunits. We compared, by western blot, the protein amount of some components of the core and CAK subcomplexes from control testes that show wild-type phenotype (*p8^+^/p8^+^*, *p8^+^/p8^−^*, *p52^+^/p52^+^*, *p52^+^/p52^EP3605^*, *p52^+^/p52^mrn^3^^*, *p52^+^/p52^mrn^5^^*) with *p8* as well as *p52* mutant testes, which show meiotic arrest phenotype. Unexpectedly, we detected p52, XPB and XPD protein levels similar to wild-type, with only a slight increase in Cdk7 in the *p8^−^/p8^−^* testes (figure 3*a*). By contrast, testes from mutant heteroallelic combinations of *p52* (*p52^EP3605^/p52^mrn^3^^* and *p52^EP3605^/p52^mrn^5^^*) showed dramatically reduced amounts (less than half) of p52, XPB and p8 compared with wild-type or heterozygotes genotypes (figure 3*b*). Furthermore, there were no significant changes in the levels of other components of the basal transcription machinery, like TBP and the RNAPII or the CAK-mediated serine 5 phosphorylation of the RNAPII-CTD (RNAPII-S_5_P-CTD) in the TFIIH mutant testes (figure 3*a*,*b*).

To better understand the effect of the *p52* mutants on the stability of the other TFIIH components, we decided to determine whether the localization of p8-ECFP was affected in the *p52^EP3605^/p52^mrn^5^^* testes, which showed reduced amounts of endogenous p52, p8 and XPB proteins. The level of p8-ECFP in testes, which was determined by immunoblotting, was unaffected in the *p52* mutant compared with p8-ECFP transgenic flies that showed normal p52 levels (figure 3*c*). However, confocal microscopy revealed that unlike to the observed enrichment in bivalent chromosomes and the nucleolus periphery of primary spermatocytes in control testes (*p52^+^/p52^EP3605^*), p8-ECFP was homogeneously distributed in the cytoplasm and the nucleoplasm in the *p52^EP3605^/p52^mrn^5^^* primary spermatocytes (figure 3*d*). By contrast, EYFP-p52 localization during spermatogenesis was unaffected in *p8^−^/p8*^−^ testes (figure 3*e*). Thus, mutations in *p52* not only affected endogenous p8 protein levels but also the p8-ECFP cellular distribution. These data showed that reduced levels of p52 have a more dramatic effect on the stability of the core TFIIH components than the absence of p8 in the fly testes. Moreover, as there was no change in TFIIH subunit levels in *p8^−^/p8^−^* other than p8, while the testes displayed a meiotic arrest phenotype similar to *p52* mutants, this suggests that a global reduction in TFIIH subunits is not the main cause for this phenotype, but it could account for the stronger penetrance observed in *p52*-affected testes. On the other hand, since the absence of p8 does not affect the stability of TFIIH or the p52 cellular localization, we cannot discard the possibility that the meiotic arrest phenotype in the *p8* mutant is generated by its interaction with other proteins or complexes besides TFIIH. Indeed, we have previously demonstrated that p8 interacts with the p18^(Hamlet)^ subunit of the SWR1 complex [16]. However, by western blot experiments we did not find any affection in p18^(Hamlet)^ levels in the *p8^−^/p8^−^* testes (figure 3*a*).

### 3.4. TFIIH mutants, with meiotic arrest phenotype, showed no reduced expression in the *aly* and *can* classes of meiotic arrest genes

Depending on their targets, most meiotic arrest genes have been classified into the following two general groups: the *always early* (*aly*) class (including *aly, comr, achi, vis, topi, mip40* and *tomb*), some of which are components of the tMAC [23,32,33,35,36] and the cannonball (*can*) class (including *can, sa, mia, rye* and *nht*), which encode for tTAFs [37,38]. The *aly* and *can* classes of genes have been proposed to be the major regulators of the testis-specific gene expression programme that allows meiotic divisions and postmeiotic spermatid differentiation. Some genes required for terminal differentiation, such as *don juan* (*dj*)*, fuzzy onions* (*fzo*) and *Male-specific RNA 87F* (*Mst87F*), are common targets between the *aly* and *can* classes. However, the transcription of some cell cycle control genes, such as C*yclin B* (*CycB*)*, boule* (*bol*) and *twine* (*twe*), only depends on *aly* class genes [39]. To investigate whether transcription in TFIIH mutant testes behaves similarly to the *aly* or *can* classes of meiotic arrest mutants, we analysed the expression of some of these terminal differentiation and cell cycle control genes in *p8^−^/p8^−^* and control testes by qPCR. Strikingly, we observed reduced expression of the *dj* and *Mst87F* transcripts, but no change in the levels of the *fzo* transcript in *p8^−^/p8^−^* compared with wild-type (*p8^+^/p8^+^*) or heterozygote (*p8^+^/p8^−^*) testes (see the electronic supplementary material, figure S1). By contrast, *p8^−^/p8^−^* testes showed slightly increased *twe* mRNA expression (see the electronic supplementary material, figure S1). These data suggest that the transcriptional defects in TFIIH mutants were not identical to those observed in mutant testes for some testis-specific transcription factors.

**Figure 4.**
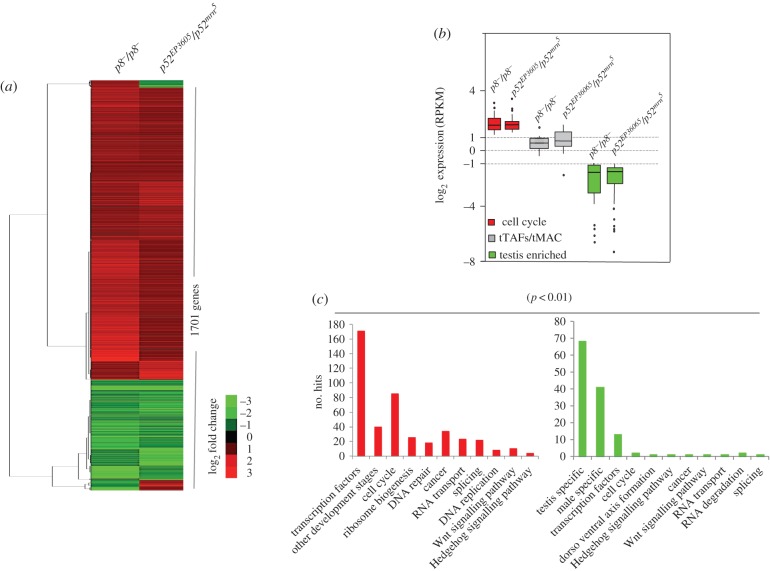
Global gene expression analyses in the *p8* and *p52* mutant testes. (*a*) Heat map of the DEGs with significant log_2_-fold change (*p* < 0.01) when compared with wild-type testes, shared between the *p8*^−^/*p8^−^* and *p52^EP3605^/p52^mrn^5^^* testes. (*b*) The box plot shows groups of genes of interest that were upregulated (cell cycle involved genes; red boxes), genes whose expression did not significantly change (*tTAFs* and *tMAC* genes; grey boxes) and genes that were downregulated (testis-enriched genes; green boxes) in TFIIH mutant testes. (*c*) Gene ontology analysis of selected functions of DEGs common between the *p8* and *p52* mutant transcriptomes when compared with wild-type (*p* < 0.01). Upregulated (red bars) and downregulated (green bars) groups of genes are shown.

Considering that TFIIH is a ubiquitously expressed central component in the basal transcription machinery for RNAPII, we aimed to analyse how TFIIH mutants affected the transcription programme required for cell differentiation in the testes. Thus, we performed global gene expression analyses (RNA-seq) of total RNA from *p8^−^/p8^−^*, *p52^EP3605^/p52^mrn^5^^* and wild-type testes. In agreement with a previously reported transcriptome analysis of *D. melanogaster* wild-type testes [30], the total number of transcripts identified in the wild-type and TFIIH mutant testes was approximately 15 000 (see the electronic supplementary material, tables S3–S5). Unexpectedly, when the two TFIIH mutants were compared to wild-type only 1701 genes were significantly differentially expressed in the *p52* and *p8* mutants (figure 4*a*; see the electronic supplementary material, table S6). The change in gene expression was very similar between both TFIIH mutants, although it was not identical, with a strongest phenotype observed in the *p52^EP3605^/p52^mrn^5^^* testes (figure 4*a*; see the electronic supplementary material, tables S7 and S8). Intriguingly, there were more upregulated (log_2_-fold change ≥ 1) than downregulated transcripts (log_2_-fold change ≤ −1) in both of the TFIIH mutants (figure 4*a*; see the electronic supplementary material, table S6). There were a few genes that were upregulated in *p8^−^/p8^−^* testes, but downregulated in the *p52^EP3605^/p52^mrn^5^^* testes and vice versa (figure 4*a*; see the electronic supplementary material, figure S2). This difference may be due to an indirect effect on gene expression between the two mutants, since the meiotic arrest phenotype is more severe in the *p52* mutant organisms.

Importantly, we found no significant changes in the transcript level of the *aly* (*tMAC*) or *can* (*tTAF*s) classes of genes in *p8^−^/p8^−^* testes (figure 4*b*). By contrast, some *can* and *aly* genes were slightly upregulated in the *p52^EP3605^/p52^mrn^5^^* testes (figure 4*b*), indicating that the meiotic arrest phenotype of the TFIIH mutant testes is not caused by decreased expression of the *aly* or *can* classes of genes.

To better understand how the misregulated genes in the TFIIH mutant testes could affect the normal cell differentiation programme in spermatogenesis, we used the demonstrated or putative function reported in the Fly Base database (http://flybase.org/) to ontologically classify the 1701 differentially expressed genes (DEGs) common to *p8^−^/p8^−^* and *p52^EP3605^/p52^mrn^5^^* testes. The DEGs were distributed in a wide range of ontological classes, suggesting that several processes were affected. In agreement with the accumulation of premeiotic primary spermatocytes observed in the TFIIH mutant testes, we found that upregulated genes included factors that regulate transcription and chromatin structure as well as genes implicated in cell cycle control (figure 4*c*; see the electronic supplementary material, tables S9 and S10). By contrast, transcripts that encode for testis-enriched proteins were notoriously reduced in the TFIIH mutant genotypes (figure 4*c*; see the electronic supplementary material, table S11). Taken together, these results show that the meiotic arrest phenotype generated by these two mutations in TFIIH subunits was not caused by the reduced expression of *aly* or *can* classes of genes, though many of their target genes were affected, suggesting that general transcription factors and testis-specific transcription factors act in coordination to regulate the specific gene programme required for normal germ cell differentiation in the testis.

### 3.5. tTAFs and TFIIH share several target genes

Meiotic arrest gene mutants other than the *aly* and *can* classes have been reported [19,28,29,40–42]. Available microarrays data were used to compare our TFIIH mutant transcriptomes with the *sa* (from the *can* class), *aly* (from the *aly* class) and the unclassified *Med22* and the *Ubi-p63E* meiotic arrest mutants. We also compared our data with the transcriptome of *bag of marbles* (*bam*) mutant testes, in which the transition from spermatogonial to primary spermatocyte is abolished and testes are enriched with over-proliferating spermatogonial cells [30]. Although these studies have been performed using different platforms, it is acceptable to compare the log_2_-fold change data obtained by RNA-seq with the geometric mean of the log_2_-fold change obtained by microarrays [43]. Therefore, the comparisons between these analyses were only at the level of what genes were upregulated or downregulated compared with the wild-type based on the significant log_2_-fold change values obtained from the GEO datasets for each mutant [19,28,29]. We focused on genes that participate in transcription, chromatin remodelling or cell cycle regulation that were upregulated and the testis-enriched genes that were downregulated in the TFIIH mutants.

**Figure 5.**
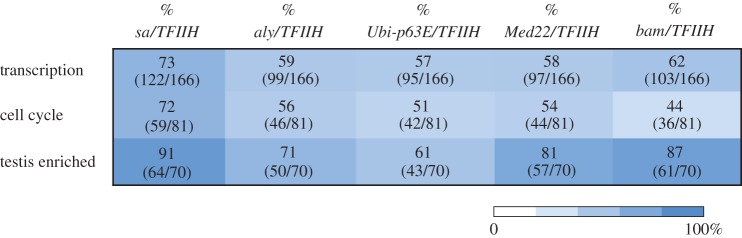
Gene expression comparison among TFIIH, *sa*, *aly, Med22*, *Ubi-p63E* and *bam* mutants. The percentage of misregulated genes involved in transcription, cell cycle or testis-enriched genes shared between the TFIIH mutants and *sa*, *aly, Med22*, *Ubi-p63E* and *bam* mutants in the *Drosophila* testis is shown. The numbers in the parentheses correspond to the number of genes misregulated in the column-correspondent mutant and TFIIH mutants between the total genes misregulated in the TFIIH mutants for each group.

In the case of the mRNAs that encode for transcription and chromatin remodelling factors, of the transcripts that increased in TFIIH mutants, 73% also increased in *sa*, 59% in *aly*, 57% in *Ubi-p63E*, 58% in *Med22* and 62% in *bam* mutant testes (figure 5). For transcripts that encode factors involved in cell cycle, of the mRNAs that are enriched in the TFIIH mutants, 72% are also increased in *sa*, 56% in *aly*, and 51%, 54% and 44% in *Ubi-p63E, Med22* and *bam* mutants, respectively (figure 5). When we compared the reduction in the transcript levels from the testis-enriched genes in the TFIIH mutants, we found that 91%, 71%, 61%, 81% and 87% are also reduced in the *sa*, *aly*, *Ubi-p63E, Med22* and *bam* mutants, respectively (figure 5). This analysis suggests that the transcriptome of the TFIIH mutant testes is more similar to the *sa* mutant and less similar to the *aly*, *Med22* and *Ubi-p63E* mutants, which also generate a meiotic arrest phenotype and accumulate primary spermatocytes. The transcriptome of the *bam* mutant that arrests spermatogenesis at the spermatogonial stage is more similar in the effect on the expression of transcription factors and differentiation genes to the TFIIH mutants than the *aly*, *Ubi-p63E* and *Med22* meiotic arrest mutants (figure 5). The strong effect on differentiation genes in the *bam* mutant is expected, since these genes are only expressed in primary spermatocytes and the *bam* mutant germ cells fail to enter this stage. On the other hand, defects in TFIIH functions may have a more general effect in gene expression than the *aly*, *Med22* and *Ubi-p63E* mutants, something that may be expected for a basal transcription factor. In summary, the TFIIH mutants displayed gene expression misregulation during spermatogenesis, which partially resemble a testis-specific TAF mutant (the *sa* mutant), suggesting that the disturbance of transcription at different levels may generate a meiotic arrest phenotype during spermatogenesis.

### 3.6. TFIIH mutant causes Pc delocalization from the nucleolus in primary spermatocytes, but not Pc-enhanced enrichment at differentiation gene promoters

**Figure 6.**
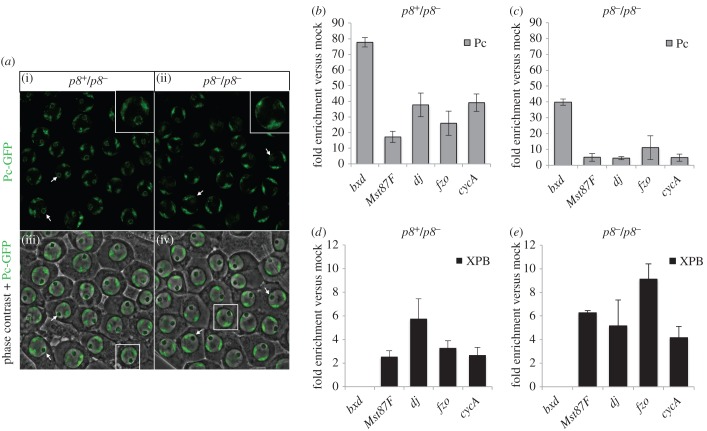
Pc is delocalized from the nucleolus in *p8* mutant testes, but does not occupy the spermatid differentiation gene promoters. (*a*) Confocal microscopy images from heterozygous (*p8^+^/p8^−^*) and homozygous (*p8^−^/p8^−^*) *p8* mutant unfixed testes show the location of Pc-GFP in mature primary spermatocytes. In *p8^+^/p8^−^* testes ((i) and (iii)), Pc is located in the bivalent chromosomes and the nucleolus periphery in primary spermatocytes. By contrast, in *p8^−^/p8^−^* testes ((ii) and (iv)), Pc is highly reduced from the nucleolus periphery in the primary spermatocytes. The arrows indicate the nucleolus in some cells. (*b*–*e*) qPCR from ChIP assays show the occupancy of (*b*,*c*) Pc and (*d*,*e*) XPB at the promoters of several spermatid differentiation genes in heterozygous (*p8^+^/p8^−^*) and homozygous (*p8^−^/p8^−^*) *p8* mutant testes. No Pc signal higher than background levels is observed in the analysed region of the promoters of *dj*, *Mst87F* and *fzo* in control or (*p8^−^/p8^−^*) testes. The *bxd* element was used as positive control and negative control for Pc and XPB binding, respectively. The *CycA* promoter is a non-target control for Pc binding. The percentage input enrichment for Pc and XPB in the analysed promoters was normalized to the mock signal detected for each sequence. Each bar shows the average and standard deviation from three biological replicates in (*b*–*d*) and two biological replicates for (*e*).

The current model for transcription regulation in the testis establishes that spermatogenesis genes are repressed by Pc in germ cell precursors until the expression of tTAFs in the nucleolus of the primary spermatocytes [20,21]. As mutants in *tTAF*s genes show de-localization of Pc from the nucleolus in primary spermatocytes, it has been proposed that part of the tTAFs mechanism of action is to sequester Pc in the nucleolus to allow the expression of the genes involved in spermatogenesis [20]. The meiotic arrest phenotype observed in TFIIH mutant testes (figure 1) and the localization of TFIIH components in the nucleolus periphery of primary spermatocytes (figure 2) prompted us to investigate whether the recruitment of Pc to the nucleolus depends on this basal transcription factor. Therefore, using the Pc-GFP transgenic fly reported previously [20,22,44], we analysed Pc localization in control (*p8^+^/p8^−^*) and *p8^−^/p8^−^* testes. Consistent with previous reports [22,44], confocal microscopy of live squashed testes showed Pc location at bivalent chromosomes and the nucleolar periphery in control primary spermatocytes (figure 6*a*(i),(iii)). By contrast, although localization in the bivalent chromosomes was unaffected, Pc was highly reduced in the nucleolar periphery of *p8^−^/p8^−^* primary spermatocytes (figure 6*a*(ii),(iv)). This suggests that irregular Pc localization is a common feature among tTAFs and general transcription factors mutants with meiotic arrest phenotypes.

The next aim was to determine whether the repression of the affected differentiation genes relied on Pc binding to their promoters in the TFIIH mutant testes. We performed ChIP experiments, followed by qPCR, to analyse the binding of Pc and the TFIIH subunit XPB at the promoters of two repressed genes (*dj* and *Mst87F*) and one unaffected gene (*fzo*) in *p8^−^/p8^−^* testes. Additionally, positive (a known target sequence in the *bxd* long non-coding RNA) and negative (the *CycA* promoter) controls for Pc binding were included in our analyses. As expected, high Pc enrichment was detected at its known canonical target (*bxd*) in both *p8^+^/p8^−^* and *p8^−^/p8*^−^ testes (figure 6*b*,*c*). On the other hand, Pc levels in the promoters of *dj, Mst87F* and *fzo* were similar to the negative control (*CycA* promoter) in the *p8^+^/p8^−^* and *p8^−^/p8^−^* testes (figure 6*b*,*c*). Therefore, Pc dissociation from the nucleolar periphery had no effect on Pc binding to the promoters of these tTAFs target genes, which were repressed in *p8^−^/p8^−^* testes. Moreover, we detected XPB occupancy at the promoter of *dj*, *fzo*, *Mst87F* and *CycA* in control (*p8^+^/p8^−^*) and *p8^−^/p8*^−^ testes, suggesting that TFIIH recruitment to the promoters of these genes was not affected (figure 6*d*,*e*). Taken together, these data indicate that the p8 subunit of TFIIH is required for Pc localization to the nucleolus in primary spermatocytes, but that Pc is not enriched at the promoters of the differentiation genes repressed in *p8^−^/p8^−^* testes.

## 4. Discussion

### 4.1. The stability of TFIIH components in the *Drosophila* testis does not rely on p8 function, suggesting an unrevealed role for p8 in transcription

Intriguingly, to date, mutations affecting only the XPB, XPD and p8 subunits have been identified in human patients afflicted with XP, CS or TTD [10–13], suggesting that mutations in other TFIIH subunits in humans may be more deleterious, or are indistinguishable from the wild-type phenotype. We have previously reported that *p52* mutant heteroallelic combinations and p8 depletion are compatible with life in *Drosophila*, but cause male sterility [15,16]. In this study, we determined that *p52* mutant or p8-depleted organisms show a meiotic arrest phenotype during spermatogenesis. This phenotype was very similar between the two mutants, though more penetrating in p52-affected testes. Further analysis of the mutant testes revealed that *p52* mutants cause an important decrease in the levels of other TFIIH core subunits, including XPB and p8. Interestingly, physical interactions among XPB, p52 and p8 have been reported [45,46]. Furthermore, p52 modulates the ATPase activity of XPB and is required for anchoring this subunit into TFIIH [7], suggesting that when p52 is affected, XPB becomes unstable and more susceptible to degradation. Strikingly, the absence of p8 in the *Drosophila* testis has no effect on the levels of other TFIIH subunits. These data seem to be against the proposed role for p8 in maintaining TFIIH stability and in contrast to the observation of lower levels of the TFIIH subunits in human cells derived from patients, who suffer from TTD-A. However, the p8 mutations described in these patients seem not to be null, and it has recently been reported that they still may interact with TFIIH [14,34]. Thus, it is possible that these mutations may generate p8 proteins that have some toxic effect when they are assembled into TFIIH, generating instability in the complex. In comparison with our analysis in the p8-depleted organism, it has been reported that XPB levels in cells derived from p8 knock-out mice, which were measured by the expression of exogenous XPB-YFP, were reduced [34]. However, it is possible that if global transcription is affected in these p8-depleted cells the expression of the transgene also might be reduced since it has been observed that the expression of transgenes is preferentially affected in comparison with endogenous genes when the basal transcription machinery is not completely functional [47]. In fact, the RNA-seq analysis from these p8-depleted early mouse embryos showed that there is a significant effect on gene expression, in particular in genes required for terminal differentiation [34], which correlates with the deregulation of transcription that we observed in the p8-depleted testes, although normal levels of other TFIIH subunits were observed.

Intriguingly, we observed that XPB is still recruited to the promoter sequences and that the global level of CAK-mediated RNAPII-Ser_5_P-CTD phosphorylation remains unaffected in p8-depleted testis. In agreement with these results, it has been recently reported that different mutations in p8 did not affect either the recruitment of TFIIH to promoters or the phosphorylation of the RNAPII-CTD in human cells [48]. Therefore, a more direct role for p8 in transcription should be considered. For instance, a possibility that might explain the effect on transcription by p8 depletion is that the interaction between p8 and p52 could be relevant for the regulatory role of p52 on XPB ATPase activity, which is required for transcription initiation by RNAPII. This is supported by recent evidence that shows that both p8 and p52 directly interact with XPB lock-N and lock-C domains [46]. Moreover, this correlates with our previous observations of enhanced transcription when p8 is added to *in vitro* transcription assays [49]. Thus, it will be relevant to analyse how XPB ATPase activity is affected by p8 and how this could affect promoter opening during transcription.

### 4.2. No Pc recruitment at spermatid differentiation gene promoters in TFIIH meiotic arrest mutants argues against the current model for transcription repression in testis

The cellular and molecular analyses of the meiotic arrest phenotype observed in the TFIIH mutant testes revealed some similarities with the main characteristics of *can* class meiotic arrest genes, though some differences can also be noted. For example, the expression of many spermatid differentiation genes, such as *Mst87F* and *dj*, were downregulated in TFIIH mutant testes, but other tTAFs targets, such as *fzo*, remained unaffected, suggesting that meiosis arrest in TFIIH primary spermatocytes was caused, at least in part, by the reduced expression levels of key spermatid differentiation gene products, some of which are common targets between tTAFs and TFIIH. Interestingly, we detected no difference in XPB recruitment at the promoters of some downregulated or unaffected spermatid differentiation genes in p8-depleted testes, suggesting that the downregulated genes identified in the TFIIH mutant testes transcriptomes represent targets that are more sensitive to TFIIH-reduced functions. Furthermore, contrary to the *aly* class but similar to the *can* class of meiotic arrest genes, the transcripts from many cell cycle regulators were not reduced in the TFIIH mutant testes. We also observed normal or even increased cyclin A and cyclin B protein levels in the TFIIH mutant testes, suggesting that the meiosis arrest in these testes was not caused by the deficiency in these key cell cycle modulators.

Interestingly, TFIIH localization in bivalent chromosomes and at the periphery of the nucleolus at the primary spermatocyte stage is very similar to the localization observed for other transcription factors, including tTAFs [22], TAF1 [50], the mediator complex [19] and the elongating form of RNAPII [22], suggesting that these regions are permissive sites for transcription. However, no evidence of transcription was detected at the nucleolar periphery of primary spermatocytes in a previous study [22]. Intriguingly, the same localization pattern has been observed for the transcriptional repressor Polycomb [22,44]. In fact, it has been proposed that nucleolar Polycomb localization in primary spermatocytes is essential to counteract its repressor role in the transcription of several tTAFs targets genes [20,21]. Intriguingly, similar to the *tTAF*s mutants, we observed Pc dissociation from the nucleolus in p8-depleted primary spermatocytes. By contrast, we did not detect Pc binding higher than the background levels observed at the non-target gene (*CycA*), in the promoter of downregulated spermatid differentiation genes in p8-depleted testes. Thus, our results agree with a recent genome-wide analysis of Pc binding sites in whole *Drosophila* testes or germline precursors that suggests that Pc is not directly involved in the regulation of tTAFs target genes during spermatogenesis, as no Pc enrichment was detected at the promoters of these genes [22]. This is further supported by a previous study that showed that the *thoc5* meiotic arrest mutant, which shows nucleolar structure disruption and concomitant Pc delocalization from the nucleolus, showed normal *Mst87F*, *dj* and *fzo* transcript expression levels, suggesting that the perinucleolar localization of Pc is not required for the expression of these spermatid differentiation genes [41]. Therefore, the evidence suggests that mutants in any of the tTAFs or components of the basal transcription machinery cause a deregulation of gene expression in primary spermatocytes that could indirectly affect Pc localization from the periphery of the nucleolus without enhancing Pc recruitment at the promoters of spermatid differentiation genes. Thus, it would be relevant to determine whether Pc localization is also affected in mutants of the recently characterized *Med22* meiotic arrest gene [19], which is a component of the mediator complex.

In conclusion, this study provides important insights about the role of TFIIH in a cell differentiation programme and how a reduction in the activities of a basal transcription factor generates specific phenotypes.

## Supplementary Material

Sup. Figures 1–2

## Supplementary Material

Sup. Tables 1–2

## Supplementary Material

Sup Tables 3–5

## Supplementary Material

Sup Tables 6–8

## Supplementary Material

Sup Tables 9–11
