# Supplementary figures and images for: Analysis of Drosophila p8 and p52 mutants reveals distinct roles for the maintenance of TFIIH stability and male germ cell differentiation
Source: Open Biol. 2016 Oct 19;6(10):160222. doi: 10.1098/rsob.160222 (PMC5090060; doi:10.1098/rsob.160222)

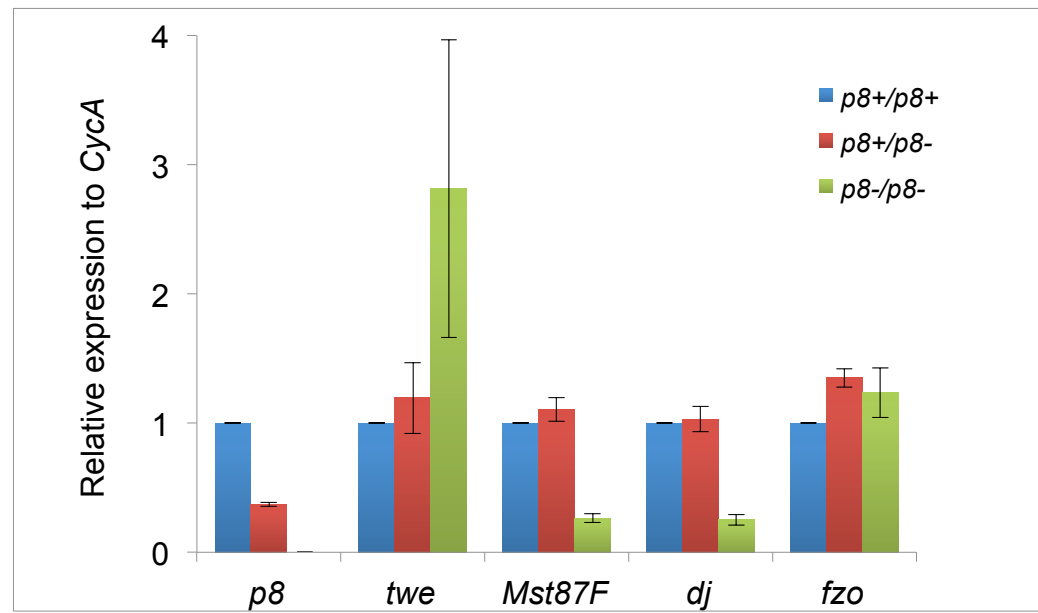

Fig S2

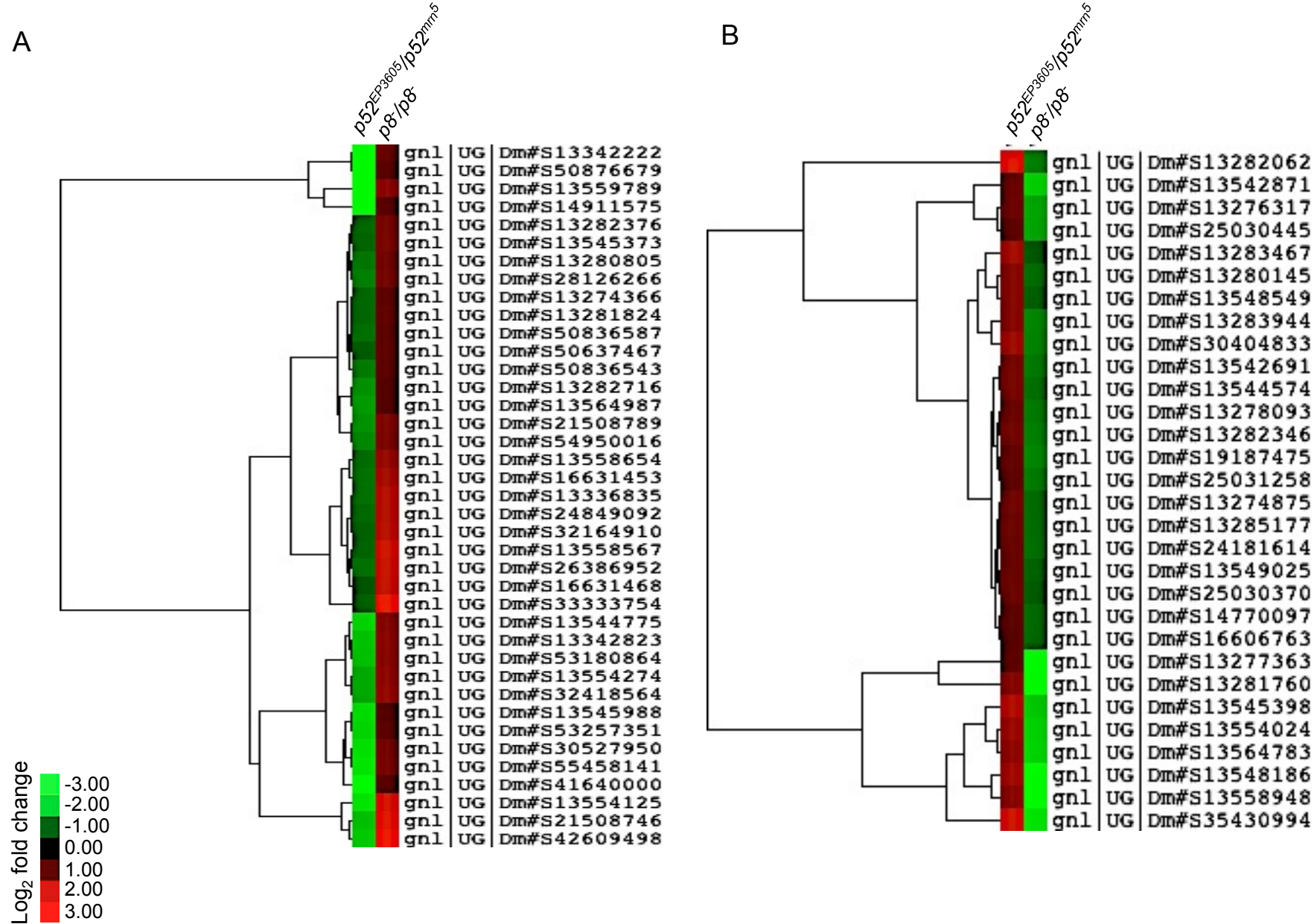

Supplement: Sup. Figures 1–2 [file rsob160222supp1.pdf]
