# Supplementary material for: Analysis of Drosophila p8 and p52 mutants reveals distinct roles for the maintenance of TFIIH stability and male germ cell differentiation
Source: Open Biol. 2016 Oct 19;6(10):160222. doi: 10.1098/rsob.160222 (PMC5090060; doi:10.1098/rsob.160222)
Supplement: Sup. Tables 1–2 [file rsob160222supp2.ppt]

## Slide 1
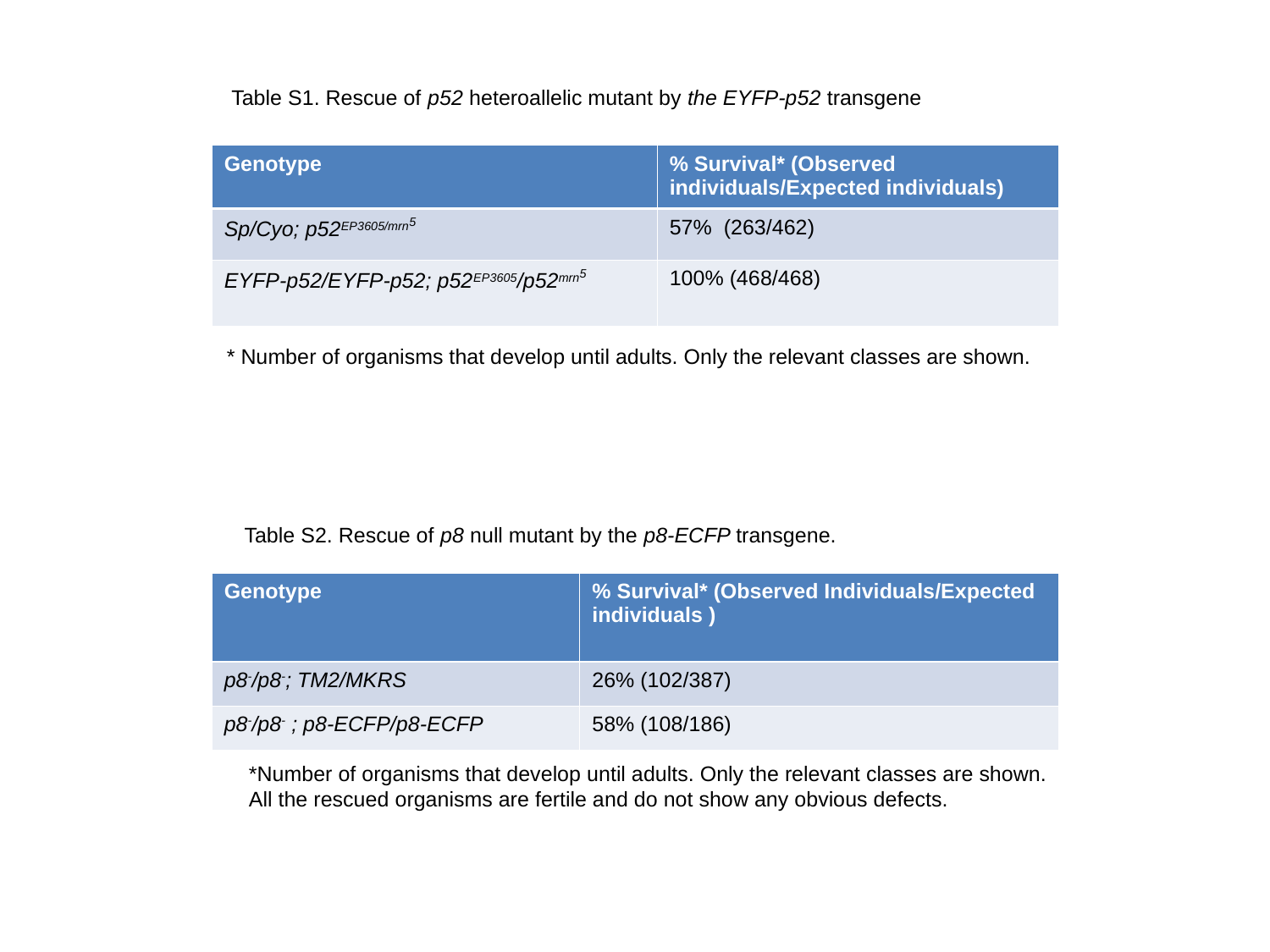

Table S1. Rescue of p52 heteroallelic mutant by the EYFP-p52 transgene
| Genotype | % Survival\* (Observed individuals/Expected individuals) |
| --- | --- |
| Sp/Cyo; p52EP3605/mrn5 | 57% (263/462) |
| EYFP-p52/EYFP-p52; p52EP3605/p52mrn5 | 100% (468/468) |
* Number of organisms that develop until adults. Only the relevant classes are shown.
Table S2. Rescue of p8 null mutant by the p8-ECFP transgene.
| Genotype | % Survival\* (Observed Individuals/Expected individuals ) |
| --- | --- |
| p8-/p8-; TM2/MKRS | 26% (102/387) |
| p8-/p8- ; p8-ECFP/p8-ECFP | 58% (108/186) |
*Number of organisms that develop until adults. Only the relevant classes are shown.
All the rescued organisms are fertile and do not show any obvious defects.
